# Combined biology-guided radiotherapy and Lutetium PSMA theranostics treatment in metastatic castrate-resistant prostate cancer

**DOI:** 10.3389/fonc.2023.1134884

**Published:** 2023-03-13

**Authors:** Mathieu Gaudreault, David Chang, Nicholas Hardcastle, Price Jackson, Tomas Kron, Michael S. Hofman, Shankar Siva

**Affiliations:** ^1^ Department of Physical Sciences, Peter MacCallum Cancer Centre, Melbourne, VIC, Australia; ^2^ Sir Peter MacCallum Department of Oncology, The University of Melbourne, Melbourne, VIC, Australia; ^3^ Department of Radiation Oncology, Peter MacCallum Cancer Centre, Melbourne, VIC, Australia; ^4^ Centre for Medical Radiation Physics, University of Wollongong, Wollongong, NSW, Australia; ^5^ Molecular Imaging and Therapeutic Nuclear Medicine, Cancer Imaging, Prostate Cancer Theranostics and Imaging Centre of Excellence (ProsTIC), Peter MacCallum Cancer Centre, Melbourne, VIC, Australia

**Keywords:** BgRT, LuPSMA, theranostics, mCRPC, PSMA

## Abstract

**Background:**

Lutetium-177 [^177^Lu]-PSMA-617 is a targeted radioligand that binds to prostate-specific membrane antigen (PSMA) and delivers radiation to metastatic prostate cancer. The presence of PSMA-negative/FDG-positive metastases can preclude patients from being eligible for this treatment. Biology-guided radiotherapy (BgRT) is a treatment modality that utilises tumour PET emissions to guide external beam radiotherapy. The feasibility of combining BgRT and Lutetium-177 [^177^Lu]-PSMA-617 for patients with PSMA-negative/FDG-positive metastatic prostate cancer was explored.

**Materials and methods:**

All patients excluded from the LuPSMA clinical trial (ID: ANZCTR12615000912583) due to PSMA/FDG discordance were retrospectively reviewed. A hypothetical workflow where PSMA-negative/FDG-positive metastases would be treated with BgRT whilst PSMA-positive metastases would be treated with Lutetium-177 [^177^Lu]-PSMA-617 was considered. Gross tumour volume (GTV) of PSMA-negative/FDG-positive tumours were delineated on the CT component of the FDG PET/CT scan. Tumours were deemed suitable for BgRT if (1) normalised SUV (nSUV), defined as the ratio of maximum SUV (SUVmax) inside the GTV to mean SUV inside a 5 mm/10 mm/20 mm margin expansion of the GTV, was larger than a pre-specified nSUV threshold and (2) there was no PET avidity inside the margin expansion.

**Results:**

In 75 patients screened for Lutetium-177 [^177^Lu]-PSMA-617 treatment, 6 patients were excluded due to PSMA/FDG discordance and 89 PSMA-negative/FDG-positive targets were identified. GTV volumes ranged from 0.3 cm^3^ to 186 cm^3^ (median GTV volume = 4.3 cm^3^, IQR = 2.2 cm^3^ – 7.4 cm^3^). SUVmax inside GTVs ranged between 3 and 12 (median SUVmax = 4.8, IQR = 3.9 – 6.2). With nSUV ≥ 3, 67%/54%/39% of all GTVs were suitable for BgRT within 5 mm/10 mm/20 mm from the tumour. Bone and lung metastases were the best candidates for BgRT (40%/27% of all tumours suitable for BgRT with nSUV ≥ 3 within 5 mm from the GTV were bone/lung GTVs).

**Conclusions:**

Combined BgRT/Lutetium-177 [^177^Lu]-PSMA-617 therapy is feasible for patients with PSMA/FDG discordant metastases.

## Introduction

Biology-guided radiotherapy (BgRT) is a novel treatment that utilises positron emission tomography (PET) signals from tumours to guide radiotherapy (1–3). Contemporary radiotherapy treatments are mainly based on image guidance provided by CT or magnetic resonance imaging (MRI). These modalities provide anatomical information which results in improved treatment planning, patient positioning, and sparing of organs at risk. PET imaging provides functional biological processes that are not visible at the anatomical scale, enhancing the determination of tissue to be targeted for treatment or to be spared as organs at risk. BgRT treatment aims to incorporate functional biological processes, determined by PET, and anatomical guidance, provided by CT and or MRI, in radiotherapy treatment (4).

BgRT is delivered with a linear accelerator equipped with PET detectors (PET-linac), currently provided by RefleXion Medical (RefleXion Medical Inc, CA, USA) (4, 5). The PET-linac is equipped with a 16-slice fan beam kVCT, dual 90° PET detectors, MV X-ray detector, 100 Hz binary multi leaf collimator and rotating ring-gantry with capacity for 60 rounds per minute. Radiation is delivered with a 6 MV flattening filter free photon beam at a nominal dose rate of 8.5 Gy/min. Some potential benefits of BgRT include real-time tracking of tumour motion and the ability to treat multiple tumours in a single session.

Prostate specific membrane antigen (PSMA) is a transmembrane protein expressed 100-1000 fold higher in prostate cancer compared with benign prostate and non-prostatic tissue. PSMA PET is associated with superior sensitivity and specificity than conventional imaging in prostate cancer (6–9, 10; 11). Due to the exceptionally high PSMA expression in metastatic castration-resistant prostate cancer, a theranostics treatment based on Lutetium-177 (^177^Lu) bound to PSMA-617 used to deliver local radiation to disease was investigated in the prospective LuPSMA clinical trial (ID: ANZCTR12615000912583) (12). In this trial, patients were first screened for eligibility based on ^18^F-fluorodeoxyglucose (FDG) and ^68^Ga-PSMA-11 PET/CT scans to confirm consistently high PSMA expression by all sites of disease. Eligible patients underwent four cycles of LuPSMA treatment. The LuPSMA clinical trial resulted in an excellent response, low toxicity profile and improved quality of life. Subsequently, phase 2 and 3 trials comparing LuPSMA with standard of care treatments in patients with metastatic castration-resistant prostate cancer were associated with superior outcomes with acceptable toxicity profiles (13).

Despite the promising data, approximately 10%-25% of screened patients were excluded from these trials due to inadequate PSMA uptake by some disease sites. These patients were not likely to benefit from radionuclide therapy. It is hypothesised that combining BgRT with LuPSMA treatment may be feasible for patients with a mix of low to high PSMA-negative/FDG-positive disease sites that would otherwise exclude them from LuPSMA therapy. In this hypothetical scenario, BgRT would be targeted towards PSMA-negative/FDG-positive disease sites whilst LuPSMA therapy would act on highly PSMA avid disease.

This study aims to determine the feasibility of BgRT to PSMA-negative/FDG-positive prostate cancer metastases, to report the anatomical distribution of PSMA-negative/FDG-positive tumours in the subset of excluded patients from the LuPSMA clinical trial, and to determine the proportion of PSMA-negative/FDG-positive tumours suitable for BgRT, in the context of a theoretical workflow for BgRT.

## Materials and methods

### Inclusion criteria

All patients enrolled in the LuPSMA prospective randomised trial (ID: ANZCTR12615000912583) at our institution but excluded due to discordance between ^68^Ga-PSMA-11 and ^18^F-FDG PET distribution were considered for inclusion (12). Patients were included in this retrospective study based on the presence of PSMA-negative/FDG-positive tumours. Images were acquired with GE Discovery PET/CT scanners (Model 690 or 710, General Electric Medical System, Milwaukee, USA).

Any FDG tumour uptake must be at the same spatial location as PSMA-avidity to be eligible for LuPSMA treatment. To verify this eligibility condition, non-physiological uptake was determined independently on the PSMA and FDG PET scans using liver-based threshold method. This exercise was completed by nuclear medicine physicians at the screening stage and resulted on a structure containing all tumour uptakes in each PET scan. These images and structures were retrospectively reviewed in this study. Images and structure sets of these patients were imported to the Eclipse treatment planning system (TPS) for gross tumour volume (GTV) delineation (v16.1, Varian Medical Systems, Palo Alto, USA). PET uptake was characterised from the standardised uptake value (SUV) normalised by body weight, to allow interpatient comparison.

### Target delineation

It was assumed that patients would undergo LuPSMA treatment from which a 100% efficacy would be achieved to PSMA avid sites. Hence, PSMA-negative/FDG-positive tumours were solely considered in this study as potential targets for BgRT. The PSMA and FDG uptake structure on the PET component of both PET/CT scan were copied to their respective CT component and fused by using the deformable registration in the TPS. The PSMA uptake structure on the CT component of the PSMA PET/CT scan was then copied to the CT component of the FDG PET/CT scan. A new structure, named FDG-PSMA, was generated from the direct subtraction of the FDG uptake structure to the PSMA uptake structure. The FDG-PSMA structure was used as a guide to delineate tumour on the CT component of the FDG PET/CT scan. An FDG-avid region may not perfectly match a PSMA-avid region because of several factors such as patient movement, respiratory motion, physiological motion, or misregistration. All potentially discordant sites identified by direct subtraction were reviewed by a radiation oncologist and a medical physicist. A subspecialist subspecialist prostate radiation oncologist subsequently delineated GTVs. Three-dimensional GTV volumes were projected on the middle slice in the coronal direction for each patient as an illustration of tumour distribution. The projection on the mid coronal section of the FDG and PSMA distribution as well as their intersection were also shown. In order to describe the distribution of tumours in the patient subset, GTVs were classified into various anatomical sites including bone, spine, lung, and nodes. The classification per site used and the number of tumours per site are shown in **Table 1**. Misregistration between the CT component and the PET component of the FDG PET/CT scan may have occurred for several reasons such as physiological or patient movement. For this reason, the FDG PET uptake was manually registered to CT contour for all tumours.

**Table 1:** Site used to classify the anatomical location of the tumours together with the number of tumours for each site.

	Site	Anatomical location	Number of tumours
1.	Bone	Pelvis, scapula, rib, pre-sacral, sternum, humerus, femur, chest, clavicle, sacrum	31
2.	Spine	C-spine, T-spine, L-spine	24
3.	Lung	Lung, mediastinum	18
4.	Nodes	Para-aortic, supraclavicular, mesorectal, paratracheal, iliac, prevascular, inguinal	16

### PET signal characterisation

BgRT requires a strong PET signal in the tumour with respect to the surrounding tissue. This condition was measured by calculating the normalised SUV (nSUV) defined as the ratio of SUVmax inside the GTV to SUVmean inside a three-dimensional shell resulting from an isotropic outer margin expansion of the GTV. Shell thicknesses of 5 mm/10 mm/20 mm were considered.

In BgRT, dose delivery is triggered from detection of annihilation photons originating from a volume called the biological tracking zone (BTZ). The BTZ was defined as the union of the GTV and the three-dimensional shell expansion. It was assumed in this study that only one tumour could be treated per BTZ. Therefore, the PET signal coming from this volume must originate from the tumour only and not from any physiological uptake in the surrounding tissue or another tumour. The PET distribution surrounding the tumour was characterised as a function of the distance from the GTV by generating consecutive 3 mm outer margin expansion of the GTV, all disjoint from each other (14, 15). The expansion was performed for distances = [3 mm, 50 mm] and SUVmax inside these shells was reported. The presence of uptake was identified through an increase in SUVmax in two consecutive pairs of shell. Manual registration, outer margin expansion, and statistics extraction were performed using the MIM Maestro software (v6.9.4, MIM inc. Cleveland, USA).

### Suitability for BGRT

Tumours were judged suitable for BgRT if (1) nSUV was greater or equal to an nSUV threshold inside the BTZ and (2) the BTZ was free of PET uptake other than the tumour. The BgRT treatment could be delivered prior to or following LuPSMA treatment. In the former case, a concordant FDG and PSMA PET avid region may be inside the BTZ and the tumour would be deemed unsuitable for BgRT under the assumption that only one tumour could be treated per BTZ. In the latter case, the concordant region would be eradicated under the 100% LuPSMA treatment efficacy assumption and the same tumour would be judged suitable for BgRT if the conditions for suitability are met. Therefore, the occurrence of the presence of a concordant avid region inside the BTZ was recorded. The analysis was performed under the two scenarios by assuming that the concordant region was absent in the latter case. This classification was performed by using nSUV thresholds ranging from 2 to 6 and BTZs generated with GTV outer margin expansions of 5 mm/10 mm/20 mm.

### Statistical analysis

Statistical differences between tumour volumes according to anatomical location and segmentation method were reported with the Wilcoxon rank sum test. The null hypothesis that medians of the distributions are similar was rejected at the 95% confidence level. Statistical correlations were determined with the Spearman correlation coefficient (r) and its associated p-value.

## Results

**Figure 1:**
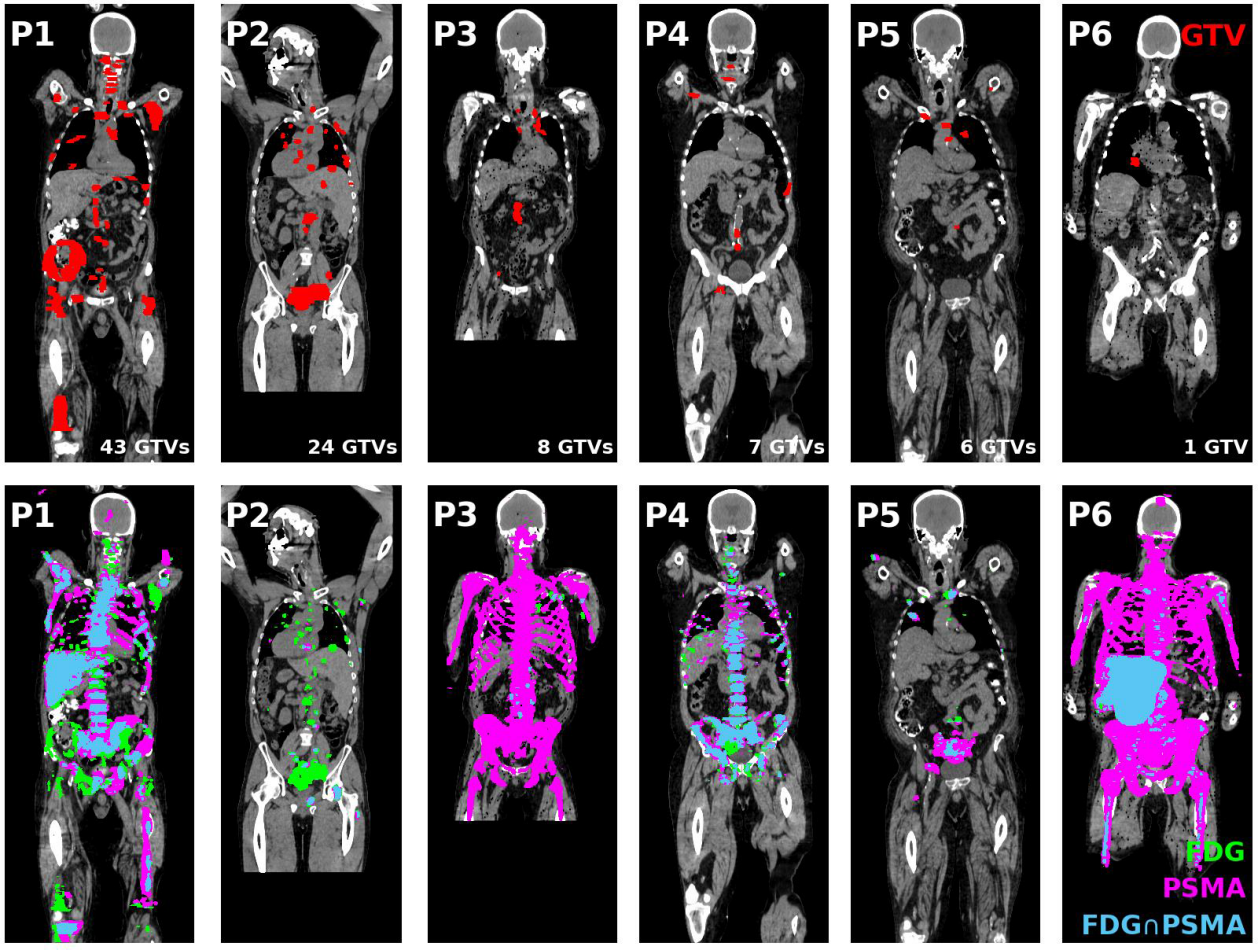
(Top tow) GTV contours (red) of all coronal slices projected on the middle coronal slice for all patients included in this study. (Bottom row) FDG (green) and PSMA (magenta) PET distribution and their intersection (blue) projected on the middle coronal slice for each patient. GTV, Gross tumour volume; FDG, ^18^F-fluorodeoxyglucose; PSMA, Prostate specific membrane antigen; PET, Positron emission tomography.

Over a cohort of 75 patients screened, 25 (33%) patients were excluded from LuPSMA treatment. From this subset of 25 patients, 7 patients (28%) were excluded due to PSMA/FDG discordance. No PSMA-negative/FDG-positive tumour was identified in one patient. Therefore, six patients were included in this study. PSMA-negative/FDG-positive regions were contoured as potential BgRT targets, resulting in 89 GTVs in total. The tumour distribution per patient as well as their associated FDG and PSMA distributions are shown in **Figure 1**. Manual registration between the CT and the PET component of the FDG PET/CT scan was minimal (median 3D shift = 0 mm). Three-dimensional shift larger than 5 mm was performed in 14 GTVs (2 patients), all located in lung.

**Figure 2:**
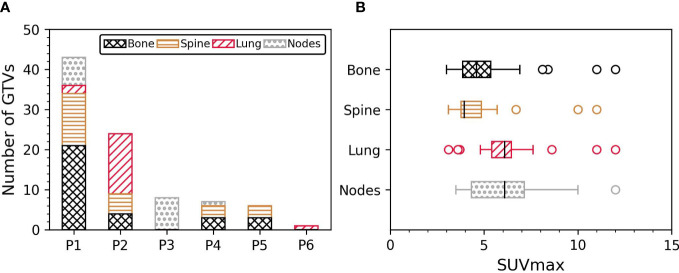
**(A)** Number of GTVs per patient and per anatomical location. **(B)** SUVmax inside the GTV per anatomical location. GTV, Gross tumour volume; SUVmax, Maximum standardised uptake value.

Four patients had less than 10 GTVs (n = 8/7/6/1 GTVs) and could be potential candidates for single session BgRT treatment. The remaining two patients had extensive disease (43 and 24 GTVs) which would require more than one BgRT session or may not be eligible for treatment based on integral/off-target radiation dose requirements. The distribution of GTVs per patient and per anatomical location is shown in **Figure 2A**. PSMA-negative/FDG-positive regions were more frequently associated with bone metastases (35%) compared with spine (27%), lung (20%) and nodal metastases (18%).

GTV volume ranged from 0.3 cm^3^ to 186 cm^3^ (median GTV volume = 4.3 cm^3^, IQR = 2.2 cm^3^ – 7.4 cm^3^). SUVmax inside GTVs was between 3 and 12 (median SUVmax = 4.8, IQR = 3.9 – 6.2). The correlation between GTV volume and SUVmax was not statistically significant (p-value = 0.3, n = 89). GTV physical volumes were similar between spine and bone metastases (p-value = 0.3), and between lung and nodal metastases (p-value = 0.7). GTV volumes of bone/spine metastases were greater than lung and nodal metastases (all p-values < 0.04 when comparing bone/spine with lung and nodes). The distribution of SUVmax per anatomical site is shown in **Figure 2B**. SUVmax was similar between lung and nodal metastases (p-value = 0.9), and between spine and bone metastases (p-value = 0.4). SUVmax in lung/nodal metastases were greater than in spine and bone metastases (all p-values < 0.04). A significant correlation between SUVmax and GTV volume was only observed in spine sites (r = 0.4, p-value = 0.03; p-values > 0.09 in other sites).

The nSUV distribution is described in **Table 2**. The nSUV distribution obtained with the 5 mm shell thickness was statistically different than the 10 mm/20 mm shell thickness (p-values < 0.03) while the distribution obtained with the 10 mm and 20 mm shell thicknesses was similar (p-value = 0.3). In particular, 69%/79%/84% of GTVs had nSUV ≥ 3 by using a margin expansion of 5 mm/10 mm/20 mm. nSUV was lower in spine sites as compared to all other sites for all margin expansions considered (median nSUV in spine = 2.4/2.6/3.0, n = 24, compared with median nSUV in other sites = 3.9/4.8/5.5, n = 65, with margin expansion 5 mm/10 mm/20 mm, p-value < 10^-4^ in all comparisons).

**Table 2:** Minimum (Min), first (Q1), second (Q2), and third (Q3) quartile, and maximum (Max) of the nSUV distribution obtained with the 5 mm/10 mm/20 mm shell thickness expansion.

		nSUV distribution5 mm shell thickness				
	n	Min	Q1	Q2	Q3	Max
All	89	1.7	2.7	3.5	4.8	10.9
Bone	31	2.4	3.1	3.8	5.0	9.2
Spine	24	1.7	2.2	2.4	3.4	6.7
Lung	18	2.6	3.7	4.1	4.7	6.3
Nodes	16	2.0	3.2	3.7	5.9	10.9
		10 mm shell thickness				
	n	Min	Q1	Q2	Q3	Max
All	89	1.8	3.1	4.0	5.7	12.0
Bone	31	2.5	3.3	4.4	6.1	10.9
Spine	24	1.8	2.3	2.6	3.5	8.3
Lung	18	3.1	4.8	5.3	5.9	7.8
Nodes	16	2.3	3.4	4.5	7.4	12.0
		20 mm shell thickness				
	n	Min	Q1	Q2	Q3	Max
All	89	2.0	3.3	4.4	6.4	12.5
Bone	31	3.0	3.8	4.5	7.0	12.5
Spine	24	2.0	2.4	3.0	3.6	9.1
Lung	18	3.4	5.7	6.3	7.3	8.6
Nodes	16	2.5	3.3	4.6	7.1	10.9

**Figure 3:**
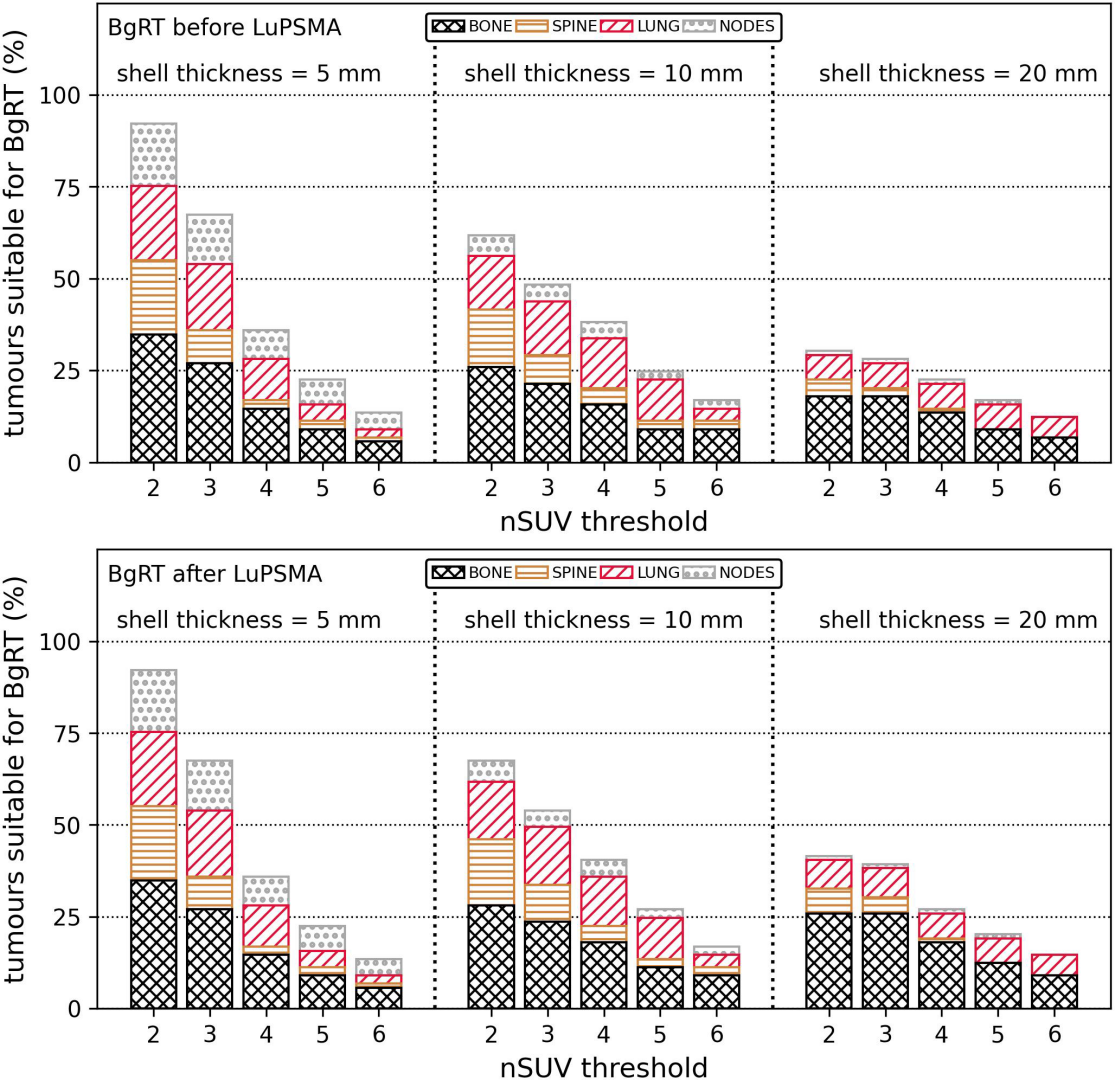
Proportion of tumours (%) suitable for BgRT (nSUV ≥ nSUV threshold and absence of PET uptake in the BTZ) depending on if the BgRT treatment was performed before (top row) or after (bottom row) LuPSMA cycle. Results were classified per anatomical site. The proportion of suitable tumours is shown for nSUV threshold between 2 and 6 and for shell thickness of 5 mm/10 mm/20 mm. BgRT, Biology-guided radiotherapy; nSUV, Normalised standardised uptake value, PET, Positron emission tomography; BTZ, Biological tracking zone.

The proximity of FDG uptake near GTVs was further evaluated. There was an FDG-avid region, either malignant or physiological, in 3%/37%/70% of all BTZs generated with the margin expansion of 5 mm/10 mm/20 mm. The proportion of tumours suitable for BgRT (nSUV ≥ nSUV threshold and BTZ free of PET uptake other than the tumour) is shown in **Figure 3** with two different scenarios whereby BgRT is delivered before or after LuPSMA cycles. The number of tumours suitable for BgRT was identical for nSUV thresholds between 2 and 6 by using a margin expansion of 5 mm in both scenarios. However, more tumours could be suitable if BgRT is delivered after LuPSMA cycles for all nSUV thresholds considered with margin expansion of 10 mm/20 mm, due to the assumption that LuPSMA treatment would completely eliminate PSMA-positive regions. If BgRT is delivered before LuPSMA therapy, 67%/48%/28% of all GTVs would be suitable for BgRT with nSUV ≥ 3 with GTV margin expansion of 5 mm/10 mm/20 mm. Under these conditions, 0%/6%/11% of all GTVs (0/5/10 tumours) were judged unsuitable for BgRT due to PET avidity originating from a concordant FDG and PSMA region within 5 mm/10 mm/20 mm from the GTV. In situations where BgRT would be performed after LuPSMA therapy, 67%/54%/39% of all GTVs would be suitable for BgRT with nSUV ≥ 3 and GTV margin expansion of 5 mm/10 mm/20 mm. The proportion of tumours suitable for BgRT was the highest in bony sites for all nSUV thresholds in all margin expansions used except in one case where the highest proportion was in lung site (nSUV = 5 with a margin expansion of 10 mm).

## Discussion

LuPSMA is a promising treatment for patient with advanced prostate cancer (16, 17, 13). However, patient with PSMA/FDG discordant prostate cancer may be excluded from this treatment and may have a poor prognosis (17, 18). A combined BgRT/theranostics treatment may offer a new option for these patients. Furthermore, BgRT to PSMA-negative/FDG-positive tumour could also be considered post LuPSMA treatment in centres that do not perform FDG PET screening to establish eligibility for the theranostics treatment. This study evaluated the feasibility of BgRT for PSMA/FDG discordant prostate cancer in combination of LuPSMA for the first time.

The feasibility of BgRT was evaluated in a subset of patients enrolled for the LuPSMA clinical trial but subsequently excluded due to PSMA/FDG discordance. This study was based on the following hypothetical clinical workflow. It was assumed that all patients would undergo LuPSMA treatment with 100% efficacy in treating all PSMA avid tumours whereas PSMA-negative/FDG-positive tumours would be treated with BgRT.

BgRT to FDG discordant disease could be provided before or after LuPSMA treatment. A greater proportion of tumours were judged suitable for BgRT if delivered after LuPSMA therapy assuming that LuPSMA would eliminate the PSMA avidity of all metastases and that only a single target could be treated within a BTZ. In particular, it was found that 67%/54%/39% of all GTVs satisfied nSUV ≥ 3 and had no PET uptake in the BTZ by using margin expansion of 5 mm/10 mm/20 mm if the BgRT treatment was performed after LuPSMA cycles. Ideal targets for BgRT were generally located in bone and lung and characterised by a strong FDG PET signal relative to the surrounding tissue.

When BgRT is delivered prior to LuPSMA therapy, the presence of a concordant PSMA/FDG metastases would prevent the successful delivery of BgRT to a large proportion of targets. The proportion of tumours suitable for BgRT may be the same in both scenarios if concordant PET region could be excluded from the BTZ (by using an asymmetrical margin expansion for instance) or if multiple tumours could be treated inside a unique BTZ.

LuPSMA therapy combined with SABR will be investigated in the prospective clinical trial POPSTAR II (NCT05560659). This workflow would be appropriate for patient with low tumour burden (smaller than 5 tumours). However, BgRT may be feasible for patients with large number of PSMA/FDG discordant tumours given the potential of BgRT to treat multiple tumours in a single session. The current study highlights the feasibility of such approach for the first time.

Tumour identification from discordant FDG and PSMA PET distribution required special consideration to manage spatial fusion. The initial threshold method used to determine the boundary for non-physiological PET uptake varies according to protocol. At our institution, three nuclear medicine physicians interpreted the images to make the final decision. The structure PSMA-FDG used to identify discordant tumour therefore has a degree of subjectivity. Moreover, the resulting PSMA avid regions and FDG avid regions may be diffuse by using a threshold method and the PSMA-FDG structure could include noisy regions often challenging to discriminate as tumours.

A 100% LuPSMA treatment efficacy was assumed which is unlikely to be achieved in all cases. Therefore, there is further opportunity to consider additional BgRT to sites of ongoing PSMA avidity due to incomplete ablation during or after cycles of LuPSMA treatment. The logistics of additional inter-cycle BgRT are not insurmountable as diagnostic PSMA PET/CT is often performed for response assessment during a treatment course. This is a line of enquiry worthy of future research.

Finally, even if a large number of GTVs were suitable for BgRT, the signal of all FDG avid tumours of a patient may not be sufficiently large to treat all of these tumours with BgRT. The remaining tumours may be treated with conventional SABR treatment, assuming that their numbers would be significantly reduced after cycles of LuPSMA therapy and BgRT treatment.

## Conclusion

Combined BgRT and LuPSMA treatment to PSMA-negative/FDG-positive tumours is feasible for patients with metastatic castrate resistant prostate cancer. This hybrid treatment may be beneficial for patients with heavy metastatic burden due to the potential ability of BgRT to treat multiple tumours in a single session efficiently.

## Data availability statement

The raw data supporting the conclusions of this article will be made available by the authors, without undue reservation.

## Ethics statement

The studies involving human participants were reviewed and approved by Peter Maccallum Cancer Centre. The patients/participants provided their written informed consent to participate in this study.

## Author contributions

All authors contributed to conception and design of the study. MG and DC analysed and interpreted the patient data. MG performed the statistical analysis. All authors contributed to the article, manuscript revision, read and approved the submitted version.
